# N-terminal prohormone B-type natriuretic peptide variability acts as a predictor of poor prognosis in patients with cardiorenal syndrome type 2

**DOI:** 10.1080/21655979.2021.2005219

**Published:** 2021-12-14

**Authors:** Mingming Ma, Qiao Luo, Xiangnan Dong, Shuang Cui, Berthold Hocher, Shufei Zeng, Wenxue Liang, Qiang Li, Xiaoyi Chen, Xin Chen, Yu Meng, Yongping Lu, Deguang Yang, Lianghong Yin

**Affiliations:** a Institute of Nephrology and Blood Purification, The First Affiliated Hospital of Jinan University, Jinan University, Guangzhou, China; b Fifth Department of Medicine (Nephrology/Endocrinology/Rheumatology), University Medical Centre Mannheim, University of Heidelberg, Heidelberg, Germany; c Department of Nephrology, Dongguan Hospital of Traditional Chinese Medicine, Dongguan, China; d Department of Cardiology, The First Affiliated Hospital of Jinan University, Jinan University, Guangzhou, China

**Keywords:** NT-proBNP variability, cardiorenal syndrome, adverse outcomes

## Abstract

This study aims to explore the effect of N-terminal pro-brain natriuretic peptide (NT-proBNP) variability (mean absolute difference of the log2 NT-proBNP level measured in hospital) on the prognosis of patients with cardiorenal syndrome (CRS) type 2. Patients with CRS type 2 were retrospectively included. The varied NT-proBNP indications were analyzed. They were NT-proBNP I(pre-treatment), NT-proBNP II(post-treatment), NT-proBNP II/I, ΔNT-proBNP, log2 (NT-proBNP) variability and mean log2 (NT-proBNP). A logistic regression model and survival curves (Kaplan–Meier analysis) were built to identify independent predictors associated with poor prognosis. The primary outcomes were major adverse renal and cardiac events. The secondary outcome was all-cause mortality. From 2012 to 2016, 136 patients were included in this study with 69 (50.7%) had high log2 (NT-proBNP) variability level. The optimal cutoff level for each NT-proBNP indication that predicts poor prognosis was calculated, and the area under curves ranged from 0.668 to 0.891 with different indications. Kaplan–Meier analysis revealed that there was significantly correlated with prevalence of primary outcomes and NT-proBNP variability. The hazard ratios (HRs) ranged from 1.67 to 6.61 with different indications. The multivariate regression analyses also identified the risk of the primary outcomes were associated with elevated NT-proBNP values, except NT-proBNP I. The odds ratio (ORs) ranged from 1.83 to 6.61 with different indications. When analyzing the relationship between NT-proBNP variability and all-cause mortality, the results were the same. NT-proBNP variability might serve as an independent predictor for poor prognosis and all-cause mortality in patients with CRS type 2.

## Introduction

Heart failure (HF) and renal dysfunction have become major and increasing public health concerns worldwide due to their high morbidities and mortality rates [1]. HF and renal dysfunction can interact with each other. The phenomenon was defined as cardiorenal syndrome (CRS). The syndrome greatly worsens prognosis and even causes death [2,3]. Five subtypes of cardiorenal syndrome (CRS) have been proposed [4,5]. CRS type 2 is the most common type that is defined as chronic cardiac insufficiency leading to progressive deterioration of chronic kidney disease (CKD) [6]. How to manage patients with CRS type 2 remains a challenge in clinical practice.

The biomarkers that reflect the true conditions of cardiac and renal disorders are important to give physicians the information when to get involved.

Serum N-terminal prohormone B-type natriuretic peptide (NT-proBNP) is predominantly synthesized and released from the ventricular cardiac myocytes in response to increased mechanical stretching or elevated filling pressures [7]. Under pathological conditions, substantial amounts of NT-proBNP are secreted into blood without processing, which can be measured in patients with HF [8–10]. NT-proBNP has been widely recognized as a powerful independent prognosticator for patients with HF [11–17].

The NT-proBNP level can be affected by various factors, such as fluid retention, age, obesity, left ventricular hypertrophy, anemia, and renal insufficiency [18]. Therefore, the bias exists in current studies making the conclusions inconsistent. Recently, a meta-analysis conducted by Tyrone *et al*. found that NT-proBNP threshold of elevation is associated with increased risk for cardiovascular (CV) and all-cause mortality in patients with end-stage kidney disease [19]. However, Hiroyuki *et al* .concluded that NT-proBNP levels did not differ during the hospitalization between patients with and without worsening renal function [20]. The inconsistent results suggest the true value of NT-proBNP in assessing CV risks in patients with renal diseases remain unclear, especially in patients with CRS type 2. Moreover, the value of individual variability of NT-proBNP during hospitalization has been scarcely explored. Hence, we hypothesized that NT-proBNP variability can be a prognosticator in patients with CRS type 2.

Therefore, the aim of the present study was to determine whether the dynamic changes in NT-proBNP levels, which we defined it as NT-proBNP variability in our study could serve as a biomarker of risk stratification and prognosis of patients with CRS type 2.

## Methods

### Study population and data collection

**Figure 1.**
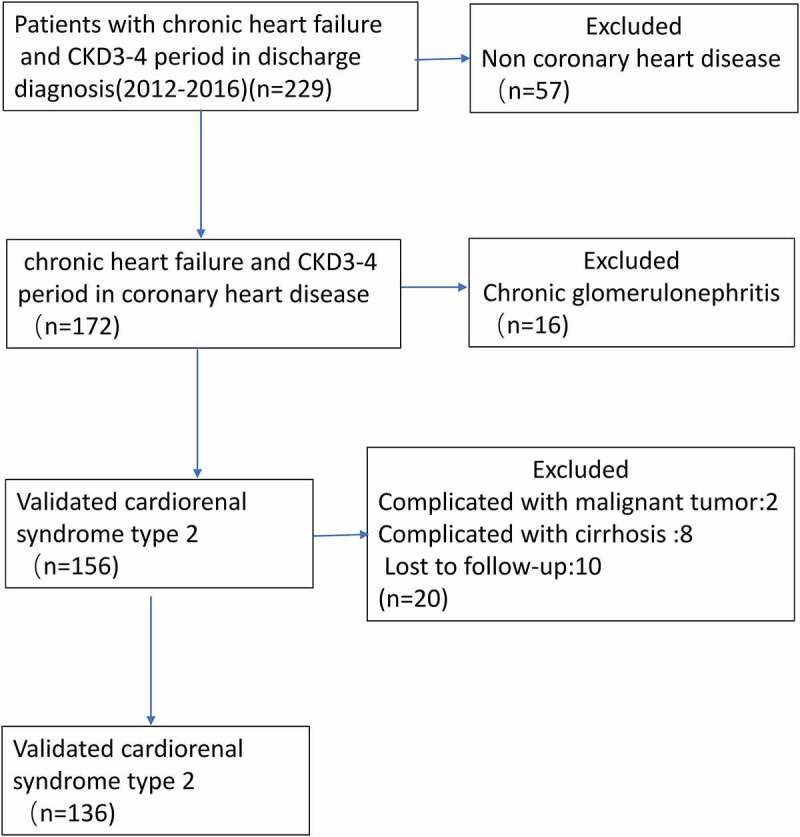
Flow diagram of included and excluded patients in the study

This study was approved by the Institutional Review Board at the First Affiliated Hospital of Jinan University (Guangzhou, China), with waived written informed consent. The retrospective study cohort consisted of 136 patients diagnosed with CRS type 2 at the Department of Cardiovascular Diseases in our hospital between 2012 and 2016. The following inclusion criteria were applied: (1) patients diagnosed with CRS type 2 based on the the 2019 American Heart association definition [21] at the time of hospitalized, and (2) patients whose full electronic medical record can be obtained. The exclusion criteria were as follows: (1) patients with stage-5 CKD requiring chronic dialysis, chronic glomerulonephritis, kidney transplantation, HF following cardiac surgery, malignant tumor, cirrhosis or multi-organ failure were excluded from analysis, and (2) patients had no record of plasma NT-proBNP concentrations before admission or during hospitalization. (3) patients who lost follow-up. The selection procedure of study participants is presented in Figure 1.

The clinical data were extracted from the hospital database, including age, sex, body height and weight, heart rate, systolic and diastolic blood pressure (SBP and DBP, respectively), hypertension history, cerebrovascular disease history, chronic obstructive pulmonary disease (COPD) history, liver disease history, atrial fibrillation (AF) history, diabetes mellitus history, hospitalization whether due to myocardial infarction, HF condition, acute kidney disease history, stroke history, malignant arrhythmias history, pharmacohistory and maintenance hemodialysis history. Laboratory parameters (i.e., hemoglobin [Hb], serum albumin [Alb], serum creatinine, electrolyte and NT-proBNP) were measured in the Central Clinical Laboratory in our hospital. All measurements were subject to routine quality controls in compliance with the laws of the People’s Republic of China.

Based on the measurement of the left ventricular eject fraction (LVEF), HF encompasses a wide range of patients, from those with preserved left ventricular ejection fraction (HFpEF), typically considered as ≥50%, to those with reduced LVEF (HFrEF), typically considered as <40%. Patients with an LVEF in the range of 40%–49% represent a ‘grey area,’ which is now defined as HFmrEF [22]. eGFR was estimated using the Modification of Diet in Renal Disease Study equation as follows: eGFR = 175 × (serum creatinine)^−1.154^ × (age)^−0.203^ × 0.742 (female). CKD was defined by an eGFR range from 15 to 60 mL/min/1.73 m^2^.

### NT-proBNP measurement

NT-proBNP variability was defined as the mean absolute difference of log2 (NT-proBNP) between each measurement during hospitalization. NT-proBNP measurements were performed using quantitative fluorescent immunoassay, (RAMP NT-proBNP test (Response Biomedical, Burnaby, British Columbia, Canada)). This is a real-time detection method, with an average time for obtaining a result of 15 minutes. A total volume of 3 mL of venous blood from each patient was withdrawn into an EDTA anticoagulation tube within 24 h or on an empty stomach in the early morning of the day after admission. All samples were directly analyzed within 2 h of blood withdrawal. A plasma NT-proBNP concentration >300 pg/mL was considered elevated [23]. NT-proBNP levels were logarithmically transformed to meet the multi-normality assumption and at least two post-baseline measurements were included in the analysis. The pretreatment NT-proBNP level on admission was labeled ‘I’, and the post-treatment NT-proBNP level at discharge was labeled ‘II’. The post-treatment NT-proBNP/ pretreatment NT-proBNP ratio were labeled as ‘II/I’. Participants were assigned into two groups based on the optimal cutoff values of the NT-proBNP I, NT-proBNP II, and NT-proBNP II/I levels, Δ NT-proBNP, log2 (NT-proBNP) variability, and the mean log2 (NT-proBNP) value determined by ROC curve analysis. The primary outcomes were major adverse renal and cardiac events (a composite of acute kidney disease, nonfatal myocardial infarction, cardiac death, stroke, maintenance hemodialysis, and repeated hospitalization for HF or malignant arrhythmias). The secondary outcome was all-cause death.

### Statistical analysis

**Figure 2.**
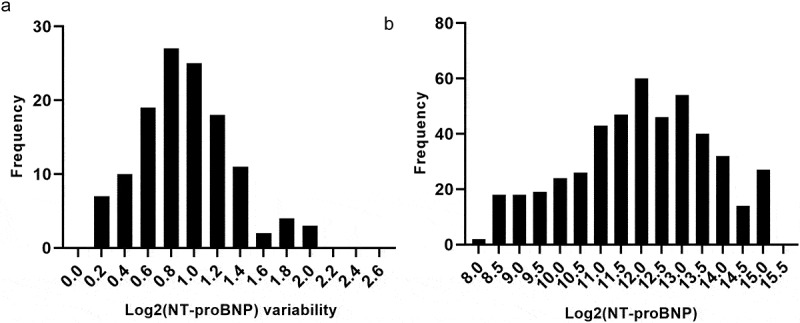
Distributions of log2 (NT-proBNP) and log2 (NT-proBNP) variability values

Continuous variables are expressed by the mean ± standard deviation or the median and interquartile ranges, while categorical variables are presented as percentages or frequencies. NT-proBNP data are presented as categorical variables or as continuous variables after natural logarithmic transformation. The distributions of the log2 (NT-proBNP) and log2 (NT-proBNP) variability values are presented in Figure 2. For univariate analysis, normally distributed values were compared between the two groups using the Student’s *t*-test, whereas abnormally distributed values were compared using the Mann–Whitney test. The Pearson χ^2^ and Kruskal Wallis tests were applied for analyses of nominal and ordinal variables, respectively. A receiver operating characteristic (ROC) curve was used to determine the greatest area under the curve (AUC) and the optimal cutoff value to predict adverse outcomes. The population was divided based on the optimal cutoff value for each marker, and survival curves were generated. Multivariate regression analysis was utilized to identify potential independent predictors of primary outcomes. Although a probability *P* < 0.05 was considered to indicate statistically significant differences, a threshold of *P* ≤ 0.1 was used for univariate analysis for the inclusion of putative risk factors into the multivariate model. All data analyses were performed using IBM SPSS Statistics for Windows, version 19.0. (IBM Corporation, Armonk, NY, USA).

## Results

This study investigated the impact of NT-proBNP variability on prognosis of patients with cardiorenal syndrome (CRS) type 2. We found that NT-proBNP variability was an independent factor with cardiorenal adverse events and all-cause mortality in patients with CRS type 2. Therefore, we proposed that NT-proBNP variability could be a prognosis predictor for patients with CRS type 2.

### Demographic characteristics

The baseline characteristics of the patients are summarized in Table 1. Of the 136 patients, 67 (49.3%) and 69 (50.7%) were assigned to the low and high log2 (NT-proBNP) variability groups, respectively, the cutoff level for log2 (NT-proBNP) was 0.62. The median number of NT-proBNP assay measurements was 5 (range, 3–32).

**Table 1.** Baseline clinical characteristics according to log2 (NT-proBNP) variability

Characteristics	All patients (n = 136)	Log2(NT-proBNP) variability < 0.62 (n = 67)	Log2(NT-proBNP) variability ≥0.62 (n = 69)	*P*
Age (years)	75.1 ± 10.8	74.3 ± 11.7	75.9 ± 9.9	0.387
Males, n (%)	67(49.3)	36 (52.2)	31 (44.9)	0.394
BMI (kg/m^2^)	23.1 ± 3.2	23.0 ± 3.3	23.2 ± 3.2	0.57
Hypertension, n (%)	106(77.9)	51 (73.9)	55 (79.7)	0.42
Cerebrovascular disease, n (%)	25(18.4)	16 (23.2)	9 (13.0)	0.12
Diabetes, n (%)	56(41.2)	24 (38.1)	32 (50.8)	0.15
COPD, n (%)	24(17.6)	10 (14.5)	14 (20.3)	0.37
AF, n (%)	50(36.7)	21 (33.3)	29 (42.0)	0.16
NYHA ≥ II, n (%)	114(83.8)	58 (84.1)	56 (81.1)	0.65
LVEF (%)	50.5 ± 10.6	52.5 ± 10.7	48.2 ± 10.2	0.02
Creatinine (mg/dL)	1.60 ± 0.71	1.66 ± 0.73	1.60 ± 0.77	0.63
eGFR (mL/min/1.73 m^2^)	44.3 ± 13.4	43.9 ± 14.3	44.6 ± 12.6	0.76
SBP (mmHg)	133.7 ± 26.5	133.8 ± 29.8	133.5 ± 23.0	0.95
DBP (mmHg)	75.9 ± 16.4	76.1 ± 18.0	75.7 ± 14.6	0.89
Hb (g/L)	117.9 ± 24.2	123.8 ± 20.9	112.6 ± 26.6	<0.01
Alb (g/L)	36.3 ± 4.8	37.7 ± 4.5	35.8 ± 4.7	<0.02
ACEI or ARB, n (%)	111(81.6)	56 (81.2)	55 (79.7)	0.83
β-Blocker, n (%)	105(77.2)	53 (76.6)	52 (75.4)	0.89
Spironolactone, n (%)	104(76.5)	54 (78.2)	50 (72.5)	0.43
Loop diuretic, n (%)	117(86.0)	60 (86.9)	57 (85.1)	0.42
PCI, n (%)	51(37.5)	24 (34.7)	27 (39.1)	0.59
Baseline NT-proBNP (pg/mL)	3278 (1655,6958)	3678 (1535,7650)	3035 (1515,6669)	0.46
NT-proBNP (pg/mL) (3 months)	3562((1497,7340)	4169 (1757,8880)	3217 (1307,6765)	0.31
NT-proBNP (pg/mL) (6 months)	4509 (2077,11,940)	5209 (2387,13,040)	4137 (1712,9323)	0.08
Baseline Log2 (NT-proBNP), (pg/mL)	8.0 ± 1.34	8.01 ± 1.40	7.99 ± 1.31	0.90
hyperkalemia	28(20.6)	10 (14.9)	18 (26.1)	0.11

Notes: Continuous variables (age, BMI, LVEF, creatinine, eGFR, SBP, DBP, Hb, Alb, log2 (NT-proBNP), and NT-proBNP) are expressed as mean (± standard deviation (and NT-proBNP as an interquartile range); categorical variables (men, hypertension, diabetes, cerebrovascular disease, COPD, liver disease, AF, cancer, NYHA ≥ II, ACEI or ARB, n, diabetes, cerebrovascular disease, COPD, liver disease, AF, cancer.

Abbreviations: ACEI, angiotensin-converting enzyme inhibitor; AF, atrial fibrillation; Alb, albumin; ARB, angiotensin II receptor blockers; BMI, body mass index; COPD, chronic obstructive pulmonary disease; DBP, diastolic blood pressure; eGFR, estimated glomerular filtration rate; Hb, hemoglobin; LVEF, left ventricular ejection fraction; NT-proBNP, N-terminal pro-brain natriuretic peptide; NYHA, New York Heart Association (classification); PCI, percutaneous coronary intervention; SBP, systolic blood pressure.

Participants in the HFrEF group generally complained of chest tightness and dyspnea, accompanied with more hospitalization times for HF. Furthermore, the participants in the HFrEF group had higher left atrial diameter (LAD), left ventricular mass index(LVMI), and left ventricular end-diastolic diameter (LVEDd) levels, but lower E/A levels than the subjects in the other two groups (HFpEF and HFmrEF groups) (*P* < 0.05). An additional file shows this in more detail [see additional file 1].

*Correlations of the NT-proBNP I, NT-proBNP II, and NT-proBNP II/I levels, log2 (NT-proBNP) variability, ΔNT-proBNP, and mean log2 (NT-proBNP) variability with the outcomes*

The patients with high log2 (NT-proBNP) variability tended to have significantly lower baseline concentrations of Hb, Alb, and LVEF (*P* = 0.01, *P* < 0.02, and *P* = 0.02, respectively). These parameters were included in multivariate analyses.

Over a median follow-up period of 22 (interquartile range = 14–36) months, a total of 62 major adverse events were identified. Patients with high *log2* (NT-proBNP) variability had a significantly higher incidence of cardiac death, repeated hospitalization for HF, malignant arrhythmias, and a higher death mortality than the lower variability group (Table 2).

**Table 2.** Incidence of primary and secondary outcomes

Log2(NT-proBNP) variability	Low (n = 67)	High (n = 69)	*P*
Primary outcomes	20	42	0.01
Acute kidney disease	6	9	0.45
Nonfatal myocardial infarction	7	6	0.73
Cardiac death	2	11	0.01
Stroke	2	3	1.0
Dialysis	4	8	0.25
Repeated hospitalization 16 for HF		29	0.02
Malignant arrhythmias 7		17	0.03
Secondary outcomes			
All-cause death	8	18	0.03

Abbreviations are as shown in Table 1

Table 3 shows descriptive statistics of the cohort grouped into patients with NT-proBNP II/I ≥ 1 and < 1. Significant differences in patients with NT-proBNP II/I ≥ 1were a lower LVEF (p = 0.01), lower Hb (p = 0.01),and a higher mean NT-proBNP(p = 0.03). Furthermore NT-proBNP II/I ≥ 1 were associated with a higher prevalence for primary events (p = 0.01),and all-cause mortality. (p = 0.03).

**Table 3.** Characteristics of the population according to NT-proBNP (II/I)

Variables	All patients, N = 136	NT-proBNP II/I > 1, N = 57	NT-proBNP II/I < 1, N = 79	*p*
Age (years)	75.1 ± 10.8	76.1 ± 10.7	74.5 ± 10.9	0.39
Males, n (%)	68 (50)	32 (56.1)	36 (45.6)	0.22
BMI (kg/m^2^)	23.0 ± 3.3	22.9 ± 2.9	23.3 ± 3.4	0.47
Diabetes, n (%)	56 (41.2)	22 (38.6)	34 (43.0)	0.60
AF, n (%)	51 (37.50)	18 (31.6)	33 (41.8)	0.23
NYHA	3.3 ± 0.74	3.3 ± 0.73	3.3 ± 0.77	1.0
LVEF (%)	49.6 ± 10.7	46.6 ± 10.96	51.7 ± 10.38	0.01
Creatinine (mg/dL)	1.6 ± 0.71	1.68 ± 0.73	1.49 ± 0.67	0.12
eGFR (mL/min/1.73 m^2^)	44.3 ± 13.4	42.4 ± 12.2	44.9 ± 14.2	0.29
SBP (mmHg)	133.7 ± 26.5	136.8 ± 28.5	131.5 ± 25.1	0.25
Hb (g/L)	117.9 ± 24.2	100.2 ± 20.6	126.2 ± 24.4	0.01
Alb (g/L)	36.3 ± 4.7	32.1 ± 3.8	38.7 ± 5.7	0.001
mean NT-proBNP (pg/mL)	4814 (2435, 11,751)	5292 (2841, 9648)	4193 (2051, 7570)	0.03
Primary events (%)	62 (45.6)	47 (82.5)	15 (18.9)	0.01
All-cause mortality	37(27.2)	21(36.8)	16(20.3)	0.03

Abbreviations are as shown in Table 1

**Figure 3.**
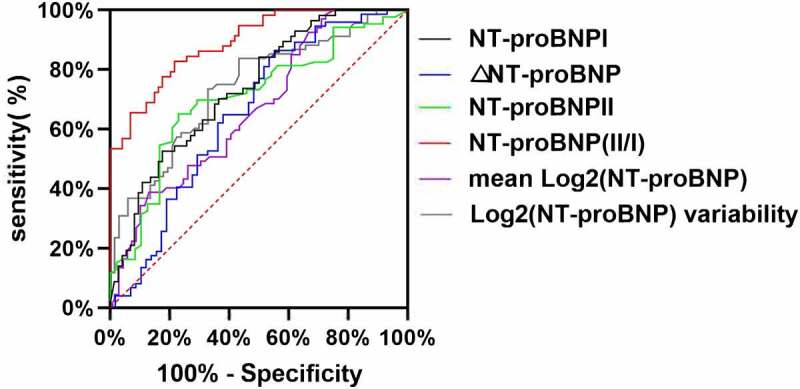
ROC curve of the NT-proBNP I, NT-proBNP II, NT-proBNP II/I, log2 (NT-proBNP) variability, ΔNT-proBNP, and the mean log2 (NT-proBNP) as a test variable and primary outcome

**Figure 4.**
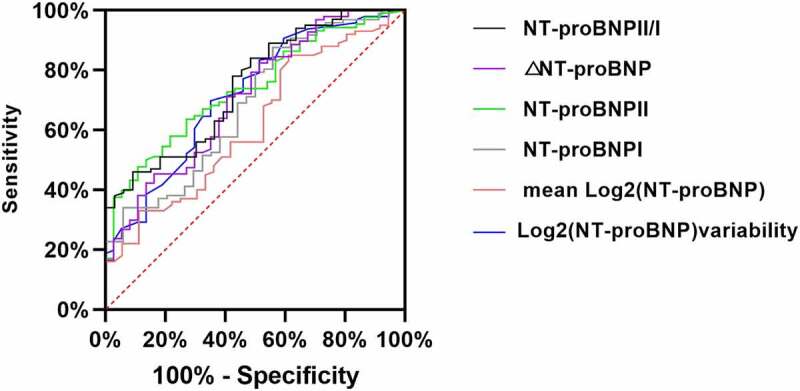
ROC curve of NT-proBNP I, NT-proBNP II, NT-proBNP II/I, log2 (NT-proBNP) variability, ΔNT-proBNP, and mean log2 (NT-proBNP) as a test variable and all-cause death

Based on the ROC curves (Figures 3, 4), we found that the AUCs of the NT-proBNP I, NT-proBNP II, and NT-proBNP II/I levels, the log2 (NT-proBNP) variability, ΔNT-proBNP, and the mean log2 (NT-proBNP) for prediction of primary outcomes were 0.705, 0.737, 0.891, 0.739, 0.724, and 0.668, respectively. The cutoff values of the NT-proBNP I, NT-proBNP II, and NT-proBNP II/I levels, the log2 (NT-proBNP) variability, ΔNT-proBNP, and the mean log2 (NT-proBNP) were 3981 ng/mL, (sensitivity, 65%; specificity, 77%); 7769 ng/mL (sensitivity, 52%; specificity, 82%); 0.93 (sensitivity, 82%; specificity, 78%); 0.62 (sensitivity, 69%; specificity, 67%); 4500 ng/mL (sensitivity, 86%; specificity, 44%); and 9.0 ng/mL (sensitivity, 39%; specificity, 87%), correspondingly (Table 4). The cutoff levels were defined as cutoff-level panels in our study.

**Table 4.** AUCs, cutoff values, sensitivity, specificity, and 95% CIs to predict primary events

Variables	Cutoff values	Sensitivity (%)	Specificity (%)	AUC	95% CI	P
NT-proBNP I	3981	65	77	0.705	0.613–0.796	0.01
NT-proBNP II	7769	52	82	0.737	0.653–0.821	0.01
NT-proBNP II/I	0.93	82	78	0.891	0.839–0.944	0.01
Log2(NT-proBNP) variability	0.62	72	65	0.745	0.676–0.828	0.01
Mean log2 (NT-proBNP)	8.91	39	87	0.668	0.578–0.758	0.01
ΔNT-proBNP	4500	86	44	0.724	0.697–0.762	0.02

Abbreviations are as shown in Table 1.ΔNT-proBNP = Absolute value of the NT-proBNP decrease = NT-proBNP I-NT-proBNP II.

**Figure 5.**
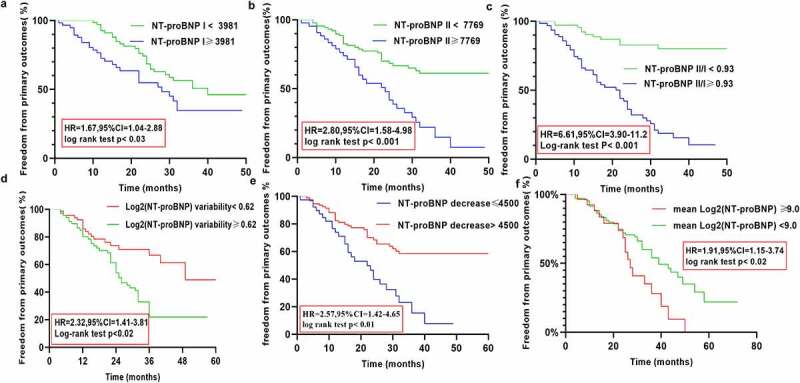
Kaplan Meier curves comparing freedom from primary outcomes vs. NT-proBNP I, NT-proBNP II, NT-proBNP II/I), log2 (NT-proBNP) variability, ΔNT-proBNP, and the mean log2 (NT-proBNP) in patients with CRS type 2

Kaplan–Meier analysis was used to assess the differences in the occurrence of the adverse outcomes until the follow-up based on the NT-proBNP I, NT-proBNP II, and NT-proBNP II/I levels, and the log2 (NT-proBNP) variability, ΔNT-proBNP, and the mean log2 (NT-proBNP) values. Kaplan–Meier analysis revealed the following results: the cutoff-level panels were significantly correlated with a higher prevalence of primary outcomes (hazard ratio [HR] = 1.67, 95% confidence ratio [CI] = 1.04–2.88, *P* = 0.03; HR = 2.80, 95%CI = 1.58–4.98, *P* < 0.001; HR = 6.61, 95% CI = 3.9–11.20, *P* < 0.001; HR = 2.32, 95% CI = 1.41–3.85, *P* < 0.02; HR = 2.57, 95% CI = 1.42–4.65, *P* < 0.01; HR = 1.91, 95% CI = 1.15–3.74, *P* < 0.02). Kaplan–Meier cumulative survival curves are presented in Figure 5.

Multivariate regression analysis was used to identify independent factors associated with primary outcomes. Based on the results of the multivariate regression analysis, we found that low Hb level, low ALB level, low LVEF, history of PCI and cutoff-level panels were independent predictors of the primary outcomes after adjustments for multiple factors, except NT-proBNP I. (Table 5). The odds ratios (ORs) for the primary outcomes in patients with elevated values of NT-proBNP II, NT-proBNP II/I, log2 (NT-proBNP) variability, ΔNT-proBNP, mean log2 (NT-proBNP), low Hb level, low ALB level, low LVEF were 2.73 (95% CI:1.61–4.77), 6.81 (95% CI:3.60–11.8), 3.35 (95% CI: 1.83–8.65), 2.47 (95% CI: 1.62–4.31), and 1.83 (95% CI:1.33–3.58), 0.97 (95% CI: 0.94–0.99),0.85 (95% CI: 0.80–0.93), 0.96 (95% CI: 0.90–0.97), respectively.-

**Table 5.** Multivariate regression analysis of the association between NT-proBNP I, NT-proBNP II level, NT-proBNPII/I, log2 (NT-proBNP) variability, ΔNT-proBNP, Mean Log2 (NT-proBNP)and the primary outcomes

Variable	Univariate *P* OR and 95% CI		Multivariate *P* ORs and 95% CI	
Hb (g/L)	0.98(0.97–0.99)	0.01	0.97 (0.94–0.99)	0.01
NT-proBNP I (pg/ml)		0.03		0.01
NT-proBNP ≥ 3981	1.67 (1.04–2.88)		1.45 (0.94–2.68)	
NT-proBNP < 3981	1		1	
NT-proBNP II(pg/mL)		0.01		0.01
NT-proBNP ≥ 7769	2.80 (1.58–4.98)		2.73 (1.61–4.77)	
NT-proBNP < 7769	1		1	
ALB (g/L)	0.92 (0.86–0.97)		0.85 (0.80–0.93)	0.01
LVEF (%)	0.52 (0.44–0.69)		0.96 (0.90–0.97)	0.01
eGFR (mL/min/1.73 m^2^	0.98 (0.96–1.01)	0.13	0.98 (0.95–1.02)	0.12
PCI		0.01		0.03
No	2.65 (1.37–5.20)		2.60 (1.35–5.32)	
Yes	1		1	
Age (years)	1.06 (1.03–1.09)	0.02	0.95 (0.91–1.02)	0.26
NT-proBNP II/I		0.01		0.02
NT-proBNP II/I ≥ 0.93	6.25 (3.45–10.8)		6.81 (3.60–11.8)	
NT-proBNP II/I < 0.93	1		1	
Log2 (NT-proBNP) Variability (pg/mL)		0.003		0.01
<0.62	1		1	
≥0.62	2.32 (1.40–3.85)		3.35 (1.83–8.65)	
ΔNT-proBNP (pg/ml)		0.01		0.02
<4500	2.57 (1.42–4.65)		2.47 (1.62–4.31)	
≥4500-	1		1	
Mean Log2(NT-proBNP), (pg/mL)		0.02		0.01
<9	1		1	
≥9	1.91 (1.15–3.74)		1.83 1.33–3.58)	

Abbreviations are as shown in Table 1

When analyzing the factors associated with all-cause mortality. We found the same parameters which is described above were independent associated with all-cause mortality after adjustments for multiple factors age, sex, and other confounding factors (Table 6), except Hb and ALB. The odds ratios (ORs) for the primary outcomes in patients with elevated values of NT-proBNP I, NT-proBNP II, NT-proBNP II/I, log2 (NT-proBNP) variability, ΔNT-proBNP, mean log2 (NT-proBNP), low LVEF were 2.05 (95% CI: 1.04–3.98), 2.13 (95% CI:1.51–3.67), 2.11 (95% CI:1.6–4.87), 1.93 (95% CI: 1.33–3.65), 2.17 (95% CI: 1.32–3.71), 2.65 (95% CI:1.23–4.56), and 0.95 (95% CI: 0.90–0.98), respectively.

**Table 6.** Multivariate regression analysis of the association between NT-proBNP I, NT-proBNP II level, NT-proBNPII/I, log2 (NT-proBNP) variability, ΔNT-proBNP, Mean Log2 (NT-proBNP)and all-cause mortality

Variable	Univariate *P* OR and 95% CI	Multivariate *P* ORs and 95% CI		
Hb (g/L)	0.78(0.41–1.39)	0.02	0.77 (0.44–1.09)	0.02
NT-proBNP I (pg/ml)		0.03		0.01
NT-proBNP ≥ 3981	2.15 (1.13–4.09)		2.05 (1.04–3.98)	
NT-proBNP < 3981	1		1	
NT-proBNP II(pg/mL)		0.01		0.01
NT-proBNP ≥ 7769	2.36 (1.04–5.36)		2.13 (1.51–3.67)	
NT-proBNP < 7769	1		1	
ALB (g/L)	0.56 (0.45–1.18)		0.55 (0.40–1.03)	0.01
LVEF (%)	0.92 (0.82–1.09)		0.95 (0.90–0.98)	0.01
eGFR (mL/min/1.73 m^2^)	0.95 (0.91–1.08)	0.33	0.98 (0.91–1.05)	0.42
PCI		0.01		0.03
No	2.85 (1.23–4.27)		2.35 (1.15–4.32)	
Yes	1		1	
Age (years)	1.05 (0.93–1.29)	0.02	1.03 (0.94–1.12)	0.20
NT-proBNP II/I		0.01		0.02
NT-proBNP II/I ≥ 0.93	2.61 (1.35–5.03)		2.11 (1.60–4.87)	
NT-proBNP II/I < 0.93	1		1	
Log2 (NT-proBNP) Variability (pg/mL)		0.02		0.03
<0.62	1		1	
≥0.62	2.03 (1.08–4.06)		1.93 (1.33–3.65)	
ΔNT-proBNP (pg/ml)		0.01		0.02
<4500	2.73 (1.12–4.04)		2.17 (1.32–3.71)	
≥4500-	1		1	
MeanLog2(NT-proBNP), (pg/mL)		0.02		0.01
<9	1		1	
≥9	2.94 (1.29–6.71)		2.65(1.23–4.56)	

**Figure 6.**
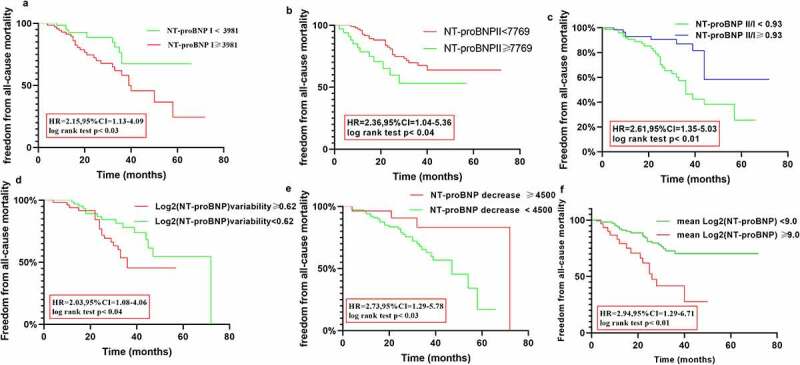
Kaplan Meier curves comparing freedom from all-cause death vs. the NT-proBNP I, NT-proBNP II, NT-proBNP (II/I), log2 (NT-proBNP) variability, ΔNT-proBNP, and the mean log2 (NT-proBNP) in patients with CRS type 2

**Figure 7.**
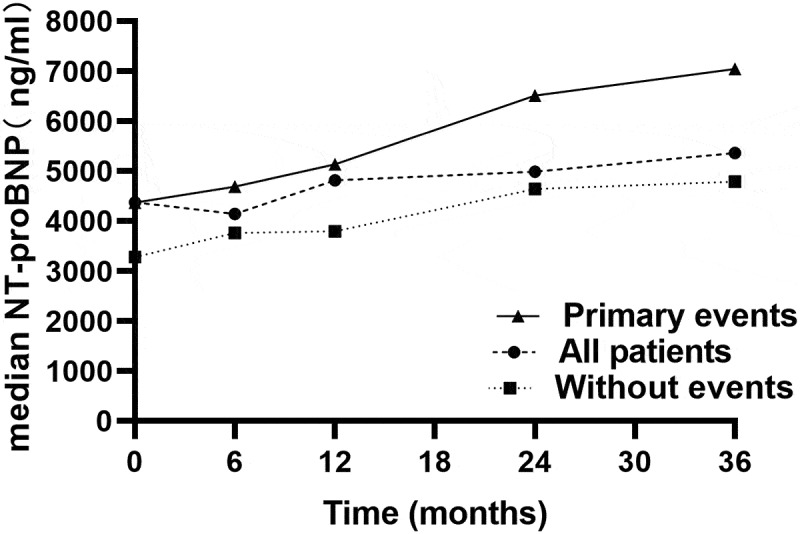
Median plasma NT-proBNP levels during the follow-up

**Figure 8.**
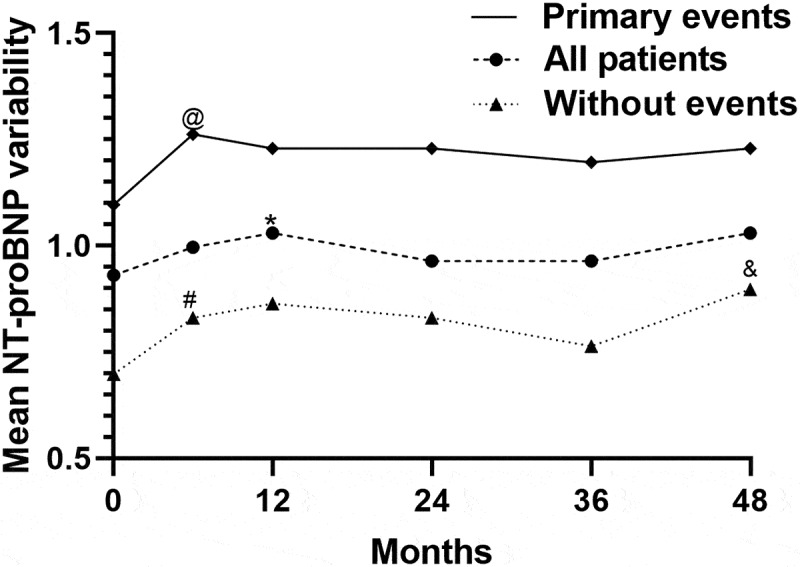
Mean log2 (NT-proBNP) variability during the follow-up

Moreover, we identified the median NT-proBNP level progressively increased in patients with primary outcomes over the follow-up period (*P* < 0.01, Figures 6–8), but the tendency was not observed with log2 (NT-proBNP) variability.

## Discussion

The management of patients with CRS type2 remains a difficulty. The biomarkers are needed to help physicians assess the patient’s condition. NT-proBNP can be used as a biomarker for the diagnosis, prognosis prediction, risk stratification, and therapeutic effect assessment of patients with HF [24,25]. For example, prior work on the relationship between circulating NT-proBNP concentrations and mortality risks reported that for every 500 ng/mL increase in the baseline NT-proBNP levels, there is a corresponding 3.8% increase in the risk of death after adjustment for other traditional risk factors [26]. Another study concluded that in patients with baseline NT-proBNP levels greater than the median value, the risk of cardiovascular and all-cause mortality was two times higher than that for those with baseline levels less than the median value [27]. Furthermore, it was observed that a dramatic decrease in the NT-proBNP level by at least 30% would result in a significant reduction in the risk of hospital readmission and death [28]. However, few studies have measured the impact of NT-proBNP level on patients with renal dysfunction. Recent studies have found that NT-proBNP alone is less predictive of future clinical adverse events in patients with advanced stages of CKD [29]. In addition, the association between the NT-proBNP level and future adverse clinical events at the time of admission was weaker than that observed at the time of discharge in patients with CKD [20,30]. A study by cheng et al. concluded that the elevated baseline NT-proBNP level is significantly associated with CV morbidity and mortality in patients with ESKD [31]. Moreover, Tyrone et al. conducted a meta-analysis with 61 studies included concluded that NT-proBNP level > 10,000 pg/mL was associated with more than 4-fold higher risks for CV mortality in patients with ESKD [18]. However, most of these studies focused on patients with CKD, and their clinical outcomes have been assessed only by NT-proBNP levels alone. Thus, the aforementioned studies established an uncertain relation of NT-proBNP level with clinical outcomes in CKD patients.

It is common that HF and CKD interact each other. Therefore, the patients with CRS type2 were included in this study. Finally, we identified the changes in the circulating levels of NT-proBNP were associated with prognosis in patients with CRS type 2. We found that the post-treatment NT-proBNP level was an independent risk factor/predictor for adverse outcomes, which is consistent with the findings of previous studies [28]. Interestingly, our present study also found that the high NT-proBNP II/I ratio and log2 (NT-proBNP) variability were independent risk factors for adverse events in patients with CRS type 2. Our current findings have extended those conclusions by Kociol et al [32]., confirming that changes in the NT-proBNP level, especially in the NT-proBNP (II/I) ratio and log2 (NT-proBNP) variability, had a higher predictive value for the prognosis of cardiorenal syndrome type2 than the NT-proBNP level at admission and discharge alone. By calculating the cutoff levels for NT-proBNPs, we provided the information that might be important for clinical practise. Our findings provide evidence for long-term involvement of risk assessment in such patients and suggest a likely application of NT-proBNP changes as novel predictor candidates of clinical outcomes and valuable indicators for risk stratification of patients with CHF complicated by CKD. The patients who had higher NT-proBNP level than the cutoff panels should undergoing more frequent follow-up evaluations. Oral medications need to be adjusted in time according to the evaluation results. Moreover, daily diet and activities need to be more restricted in these patients.

However, our outcomes are contrary to that of Hanlon et al., who found that the changes in the NT-proBNP values were relatively large in stable HF patients, accounting for up to 50% on a weekly basis [32]. Nevertheless, Cortés et al. reported good stability of the NT-proBNP level in patients with stable HF during a 24-month follow-up period; their findings also revealed that the variations in NT-proBNP concentrations exceeding 25% might indicate further pathophysiological changes in the 24-month follow-up period [33]. A possible explanation of the opposite results in these studies may be that the pathophysiological and clinical symptoms change lagged behind those of NT-proBNP change, the follow-up time was not long enough. Therefore, the application of log2(NT-proBNP) variability for stable CRS type2 patients facilitates the interpretation of early changes in clinical conditions. Furthermore, the elevated levels of NT-proBNP are associated with a variety of cardiac and noncardiac causes [34]. Therefore, the threshold value of log2 (NT-proBNP) variability that can be used to identify the presence of a change in early decompensation or worsening renal function remains unclear. The multi-point dynamic detection of NT-proBNP other than spot detection is more likely to reflect more accurately the real changes over time in disease progression. The improved measuring modality can provide more valuable and precise prognostic information. However, our findings need to be confirmed in further studies that would also reveal the underlying mechanisms in greater detail.

Although our hypotheses were supported statistically, there are limitations to this study concerning the interpretations of these findings. First, this study was based on data from a single source and the sample size was small, and thus the power of samples to determine the predictive value of NT-proBNP variability of clinical outcomes was limited. Second, the present investigation was retrospective, and the reasons for the dropping-out of some patients were missing, which could have led to biased results. Third, other biomarkers, such as serum troponins and cystatin C, which are considered to be capable of predicting outcomes, were not assessed here. Therefore, our findings should be considered as preliminary, and a larger sample with a longitudinal design would be required to elucidate the causality between NT-proBNP variability and adverse outcomes in patients with CRS type 2.

### Conclusion

Our study showed that high NT-proBNP variability was an independent factor associated with poor prognosis in patients with CRS type 2. If further confirmed, NT-proBNP variability would provide more valuable information of patients with CRS type 2 than measurements of NT-pro BNP level alone.

## Supplementary Material

Supplemental Material

## Figures and Tables

**Table:** 

Abbreviation	
NT-pro-BNP	N-terminal pro-brain natriuretic peptide
CRS	Cardiorenal syndrome
CHF	Chronic heart failure
CKD	Chronic kidney disease
ROC	Receiver operating characteristic
ESKD	End-stage kidney disease
SBP and DBP,	Systolic and diastolic blood pressure
COPD	Chronic obstructive pulmonary disease
AF	Atrial fibrillation
Hb	Hemoglobin
Alb	Albumin
CT	Computed tomography
LVEF	Left ventricular eject fraction
HFpEF	Heart failure with preserved left ventricular ejection fraction
HFrEF	Heart failure with reduced left ventricular ejection fraction
HFmrEF	Heart failure with mid-range ejection fraction
eGFR	Estimation glomerular filtration rate
AUC	Area under the curve
NYHA	New York Heart Association
PCI	Percutaneous coronary intervention
HR	Hazard ratio

## Data Availability

Data are available from the corresponding authors upon reasonable request.

## Research highlights:

- NT-proBNP variability is an independent factor with adverse renal and cardiac events
- NT-proBNP variability is an independent factor with all-cause mortality
- NT-proBNP variability is a more suitable prognosis indication than single measurement

## Study approval

The protocol of this retrospective study was approved by the Institutional Ethical Review Board of Jinan University (Guangzhou, China) and conducted in accordance with the tenets outlined in the Declaration of Helsinki.
