# Supplementary material for: N-terminal prohormone B-type natriuretic peptide variability acts as a predictor of poor prognosis in patients with cardiorenal syndrome type 2
Source: Bioengineered. 2021 Dec 14;12(2):12407–19. doi: 10.1080/21655979.2021.2005219 (PMC8810077; doi:10.1080/21655979.2021.2005219)
Supplement: Supplemental Material [file KBIE_A_2005219_SM9969.docx]

Supplementary materials:

Table S1:Comparison of clinical symptom and Doppler echo characteristics according to ejection fraction

| **Variables** | **HFrEF( <40%)**  **N=64** | **HFmrEF(40 to <50%)**  **N=30** | **HFpEF(≥50%)**  **N=42** |
| --- | --- | --- | --- |
| chest tightness | 52（81.1）^○^ | 22（73.3） | 24（57.1） |
| dyspnea | 55（85.9）^◆△^ | 20（66.6） | 27（64.2） |
| duration (months) | 3.5(0.5,8) | 5.0(0.7,9) | 4.5 (1,8) |
| previous HF hostpialisation | 58(90.6) | 28(93.3) | 35(83.3) |
| LAD(mm) | 43.8±8.7^△^ | 40.8±6.2 | 40.7±4.2 |
| E/A | 0.71±0.19^◇△^ | 0.82±0.18 | 0.83±0.21 |
| LVMI(g/m^2^) | 171±45.2^◇○^ | 144±41.3^△^ | 124±29.2 |
| LVEDd (mm) | 61.4±9.2^○◇^ | 52.6±7.5^△^ | 49.0±3.7 |

Abbreviations: LVEDd, left ventricular end-diastolic diameter; LAD,left atrial diameter; LVMI, left ventricular mass index.

△p < 0.05 vs. HFpEF. ◇p < 0.01vs. HFmrEF, ○p < 0.01 vs. HFpEF,◆ p < 0.05 vs. HFmrEF.

As shown in table S1, individuals in the HFrEF were generally Chest tightness, more often dyspnea and had a history of previous HF hostpialisation. Furthermore, individuals in the HFrEF had higher LAD, LVMI and LVEDd levels, while they had lower E/A lev­el in comparison to subjects in the lower two group (p<0.05).
